# Adaptive support ventilation as an acceptable mode to prevent airflow limitation, air entrapment, dynamic hyperinflation and patient-ventilator dyssynchrony

**DOI:** 10.1186/2197-425X-3-S1-A826

**Published:** 2015-10-01

**Authors:** A Taha, A Shafie, Y Lavoie, H Hubert, R Marktanner

**Affiliations:** Division of Adult Cardiac and Transplant Critical Care, Critical Care Department, Sheikh Khalifa Medical City Managed by Cleveland Clinic, Abu Dhabi, United Arab Emirates; Division of Respiratory Therapy, Respiratory Therapy Department, Sheikh Khalifa Medical City Managed by Cleveland Clinic, Abu Dhabi, United Arab Emirates; Division of Respiratory Therapy, Respiratory Therapy Department, Abu Dhabi, United Arab Emirates

## Introduction

Adaptive support ventilation (ASV) is a dual control mode, using measured dynamic compliance and time constant, with an automated adjustment of tidal volume and respiratory rate combined to meet the preset minute ventilation. The purpose of this study was to evaluate the effectiveness of (ASV) in restoring air flow stability, decrease work of breathing and improving dys-synchrony in ventilated patients with variable airway resistance.

## Methods

20 ICU patients showing air entrapment, Dynamic Hyperinflation and patient-ventilator dyssynchrony were enrolled between january 2014 and March 2015. Data was collected 48 hours after switching to ASV, which was applied with the same protocol. All enrolled patients were hemodynamically monitored. A retrospective analysis of this data was performed.

## Results

Completed data sets were obtained from 20 patients. the average peak airway pressure was 28 cm H2O. Peak airway pressure decreased 19% (p < 0.01), flow time curves improved by 80% (p < 0.001), with decrease work of breathing (p < 0.015) over short time from starting the patient on ASV mode.

## Conclusions

In our patient series, ASV significantly improved dys-synchrony by using measured dynamic compliance and time constant cycle by cycle with subsequent optimization of flow time curves with less use of sedation and neuromuscular blockage. Furthermore, this strategy improved hemodynamics and facilitated weaning from MV. Therefore, our data suggests, that this ventilation modality has favorable results and appears to be an effective tool in patients with air flow resistance, who develop air entrapment, Dynamic Hyperinflation and patient-ventilator dyssynchrony during their ICU stay.
